# Unveiling the Enigma: A Confluence of Two Case Reports for the Negotiation of Mid-Mesial Canals

**DOI:** 10.7759/cureus.51746

**Published:** 2024-01-06

**Authors:** Swayangprabha Sarangi, Manoj Chandak, Shweta Sedani, Paridhi Agrawal, Mrinal Nadgouda, Unnati Shirbhate

**Affiliations:** 1 Department of Conservative Dentistry and Endodontics, Sharad Pawar Dental College, Datta Meghe Institute of Higher Education and Research, Wardha, IND; 2 Department of Periodontics, Sharad Pawar Dental College, Datta Meghe Institute of Higher Education and Research, Wardha, IND

**Keywords:** mid-mesial canal, magnification, obturation, mandibular molar, endodontics

## Abstract

Mandibular molars display a wide spectrum of intricate anatomical variations among the Indian population. This case report details the diagnosis and successful endodontic management of a mid-mesial canal in the mandibular first molar tooth, emphasizing the importance of radiographic imaging and meticulous instrumentation. The patient presented with symptoms of irreversible pulpitis that led to accurate relief of symptoms after shaping and cleaning protocols were followed. This case series discusses the challenges encountered during canal negotiation, cleaning, shaping, and obturation, providing insights into the complexities associated with mid-mesial canals.

## Introduction

The success of endodontic treatment is governed by satisfactory debridement of the canal, followed by the achievement of a hermetic seal and three-dimensional filling of the radicular space. The reason for failure of treatment even after achieving the highest standards of protocols in irrigation, using the best endodontic sealers, and incorporating the best obturating material lies in the failure to identify the canal configurations in molar teeth. Iatrogenic failure in the identification of canals is primarily due to the inadequacy of knowledge and lack of clinical skills to negotiate it. The root canal anatomy is itself a complex network of pathways, and proper cleaning and shaping methods are needed to gain access and visibility through it. Prior radiographic judgment and the presence of complexities are prerequisites needed for the assessment of outcomes following root canal procedures [1].

An array of anatomical variations exist with the mandibular first molar, such as the incidence of numerous canals in mutually mesial and distal roots in the form of a mid-mesial canal or a mid-distal canal, additional roots in the form of radix entomolaris and radix paramolaris, and C-shaped canal configurations [2-5]. According to the available literature, the prevalence rates of mid-mesial canals vary around 46.2%, whereas some races show occurrence rates of up to 53.8% also. In 1981, Pomeranz et al. categorized mid-mesial canals into three different varieties, namely, fin, confluent, and independently present canal types [2].

The intricate anatomy of root canal systems poses challenges for endodontic practitioners, where the presence of mid-mesial canal in mandibular molars is often overlooked. This case report highlights the need for eyeing supplementary canals and infrequent canal morphologies, as awareness of their presence serves as an eye-opener, enabling dental practitioners to minister endodontic procedures that otherwise might have resulted in failures. The significance of using additional aids in magnification, such as dental operating microscopes and dental loupes, as well as the utilization of enhanced imaging modalities, helps in the identification and negotiation of prevalent anomalies [6].

## Case presentation

Case 1

**Figure 1:**
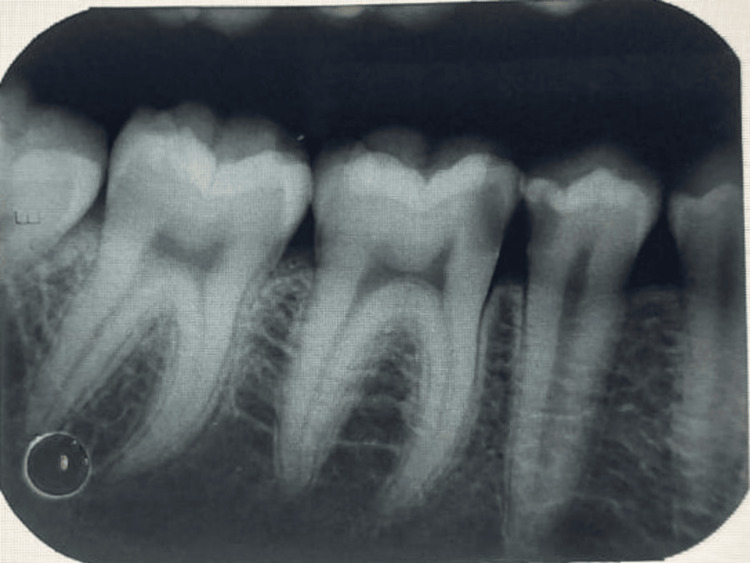
Preoperative intraoral periapical radiograph of the mandibular right first molar tooth

A 27-year-old female patient reported to the outpatient department with the chief complaint of pain and swelling in the lower right back region of the jaw for 15 days where she was clinically examined before starting the procedure, and written informed consent was obtained, after which local anesthesia containing lignocaine (2%) with 1:100,000 units of epinephrine was administered. Priorly, a diagnostic intraoral periapical radiograph demonstrated the extension of caries up to the level of the involvement of the pulp, which needed an endodontic intervention to be performed (Figure 1).

Following standard protocols of rubber dam isolation, conventional class I endodontic access cavity preparation was done using a size 2 endodontic access bur (Dentsply Sirona, Charlotte, NC).

Further extension and refinement of the access cavity was done using Endo Z bur (Dentsply Sirona). Pulpal cavity assessment was done with an endodontic explorer, while patency was established with a size 10 Kerr file (Mani, Japan). Initially, four canals were negotiated, two of which were identified mesially and two located distally. To aid visibility and provide superior amplification, a dental loupe of 3× magnification was used, and the confirmation of the same was carried out under a dental operating microscope (Labomed Prima DNT Dental Surgical Microscope, Los Angeles, CA).

**Figure 2:**
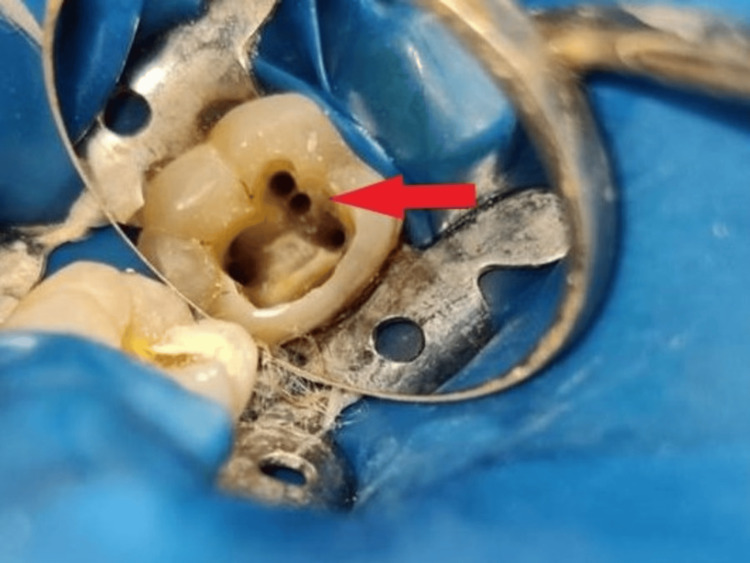
Access opening with the mandibular right first molar with the red arrow indicating the presence of the mid-mesial canal

A tiny bleeding spot was recognized in between the mesiobuccal and mesiolingual orifice openings. Using ultrasonic tips of size Start-X #1 and Start-X #3 (Start-X, Dentsply Sirona), exploration of mid-mesial orifice openings was done and processed with a 10K file (Figure 2).

**Figure 3:**
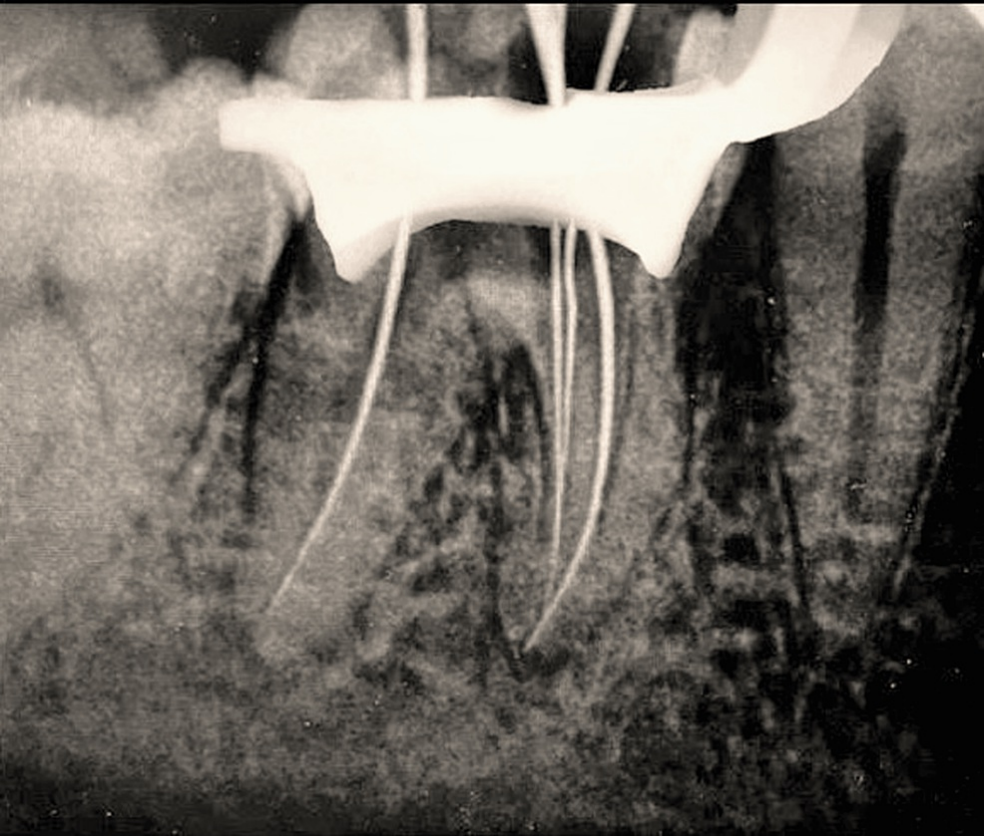
Working length determination in the mandibular molar measured using a 10 Kerr file (Mani, Japan)

Determination of working length was done using J Morita Root Zx Mini Apex Locator (Japan) (Figure 3). The working lengths of the four canals were 20 mm for the mesiobuccal canal and mesiolingual canal, 21 mm for the mid-mesial canal, and 21 mm for the distal canal.

A sum total of four separated orifices were discovered; i.e., three mesial (mesiobuccal, mesiocentral, and mesiolingual) and one distal were negotiated. Due to the wide width of all canals containing the mid-mesial canal, shaping and cleaning were performed with the crown-down method using a ProTaper Universal NiTi rotary endodontic files (Dentsply Sirona) up to size F2 (6%) for all canals.

**Figure 4:**
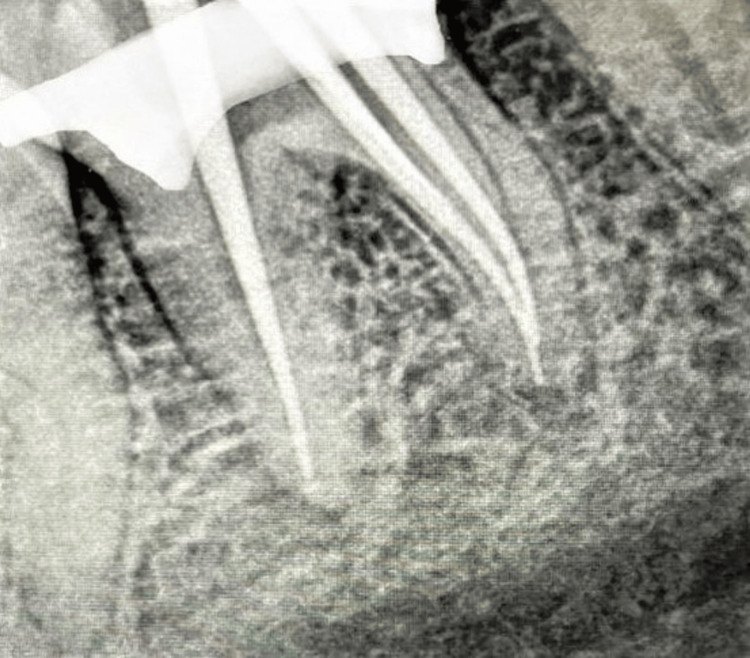
Mastercone fit assessment done with gutta-percha cones of size 6% taper (Dentsply Sirona) in the mandibular molar

Cleaning was performed using 3% NaOCl solution (Vishal Dentocare Private Limited, Ahmedabad, Gujarat, India) and 17% NeoEDTA gel using passive ultrasonic irrigation (Neoendo, Orikam Healthcare Private Limited, Gurgaon, Haryana, India). The mid-mesial canal was independently present from the orifice to the apex of the mesial root as was revealed by taking radio-visiographs under different angulations. The root canal was dried with the help of paper points and obturated with 6% gutta-percha cones (Dentsply Sirona) using the technique of warm vertical compaction along with epoxy resin sealer (AH Plus sealant, Dentsply Sirona) (Figure 4).

**Figure 5:**
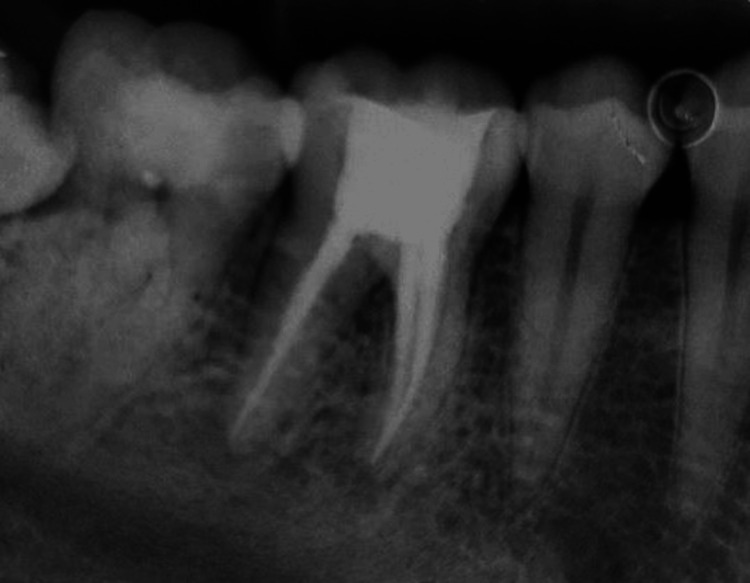
Obturation and post-endodontic restoration with composite resin

Post-obturation radiographs showed that the mid-mesial canal was fused with the mesiobuccal canal in the middle third of the root. Restoration of the prepared coronal access cavity was done using composite resin (3M™ ESPE Filtek™ Bulk Fill, 3M, St. Paul, MN) (Figure 5).

Case 2

**Figure 6:**
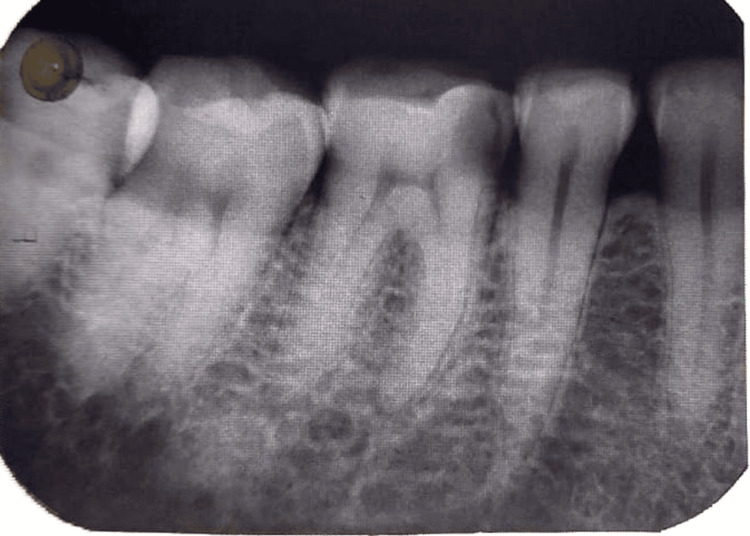
Preoperative intraoral periapical radiograph of the mandibular right first molar tooth

A 28-year-old male patient reported to the outpatient department of the dental OPD with pain and discomfort in the right mandibular back region of the jaw over the past two weeks. The patient reported taking analgesics to relieve the pain that he was experiencing. Clinical and radiographic findings concluded that the tooth was associated with symptomatic irreparable pulpitis. Caries excavation was done following prior radiographic assessment, upon which extension of the carious pathology was seen invading the pulp in the right mandibular first molar number 46 (Figure 6).

**Figure 7:**
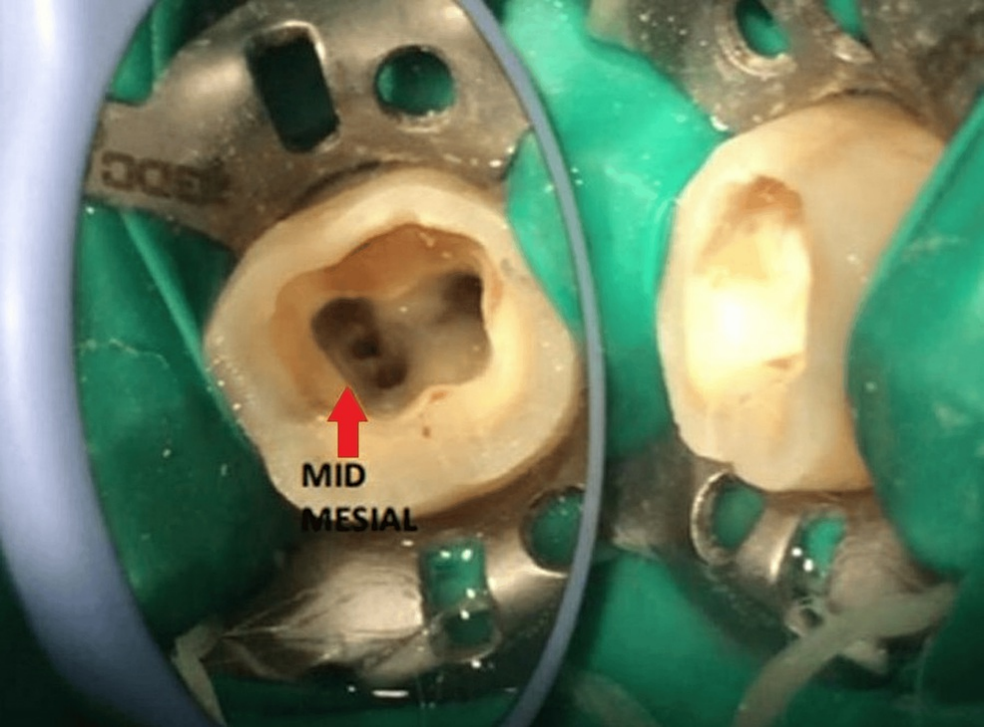
Access opening with the mandibular right first molar indicating the mid-mesial canal

**Figure 8:**
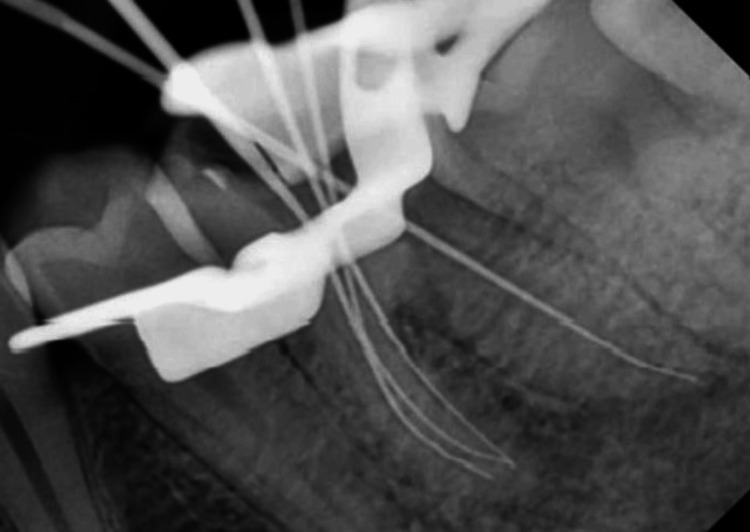
Working length determination in the mandibular molar measured using a 10 Kerr file (Mani, Japan)

The treatment protocol followed was as described in the first case. A dental loupe of 3× magnification was used, and the confirmation of the same was carried out under a dental operating microscope (Labomed Prima DNT Dental Surgical Microscope). Negotiation of four different orifices was done: three mesially (mesiobuccal, mid-mesial canal, and mesiolingual) and a distal canal (Figure 7 and Figure 8).

**Figure 9:**
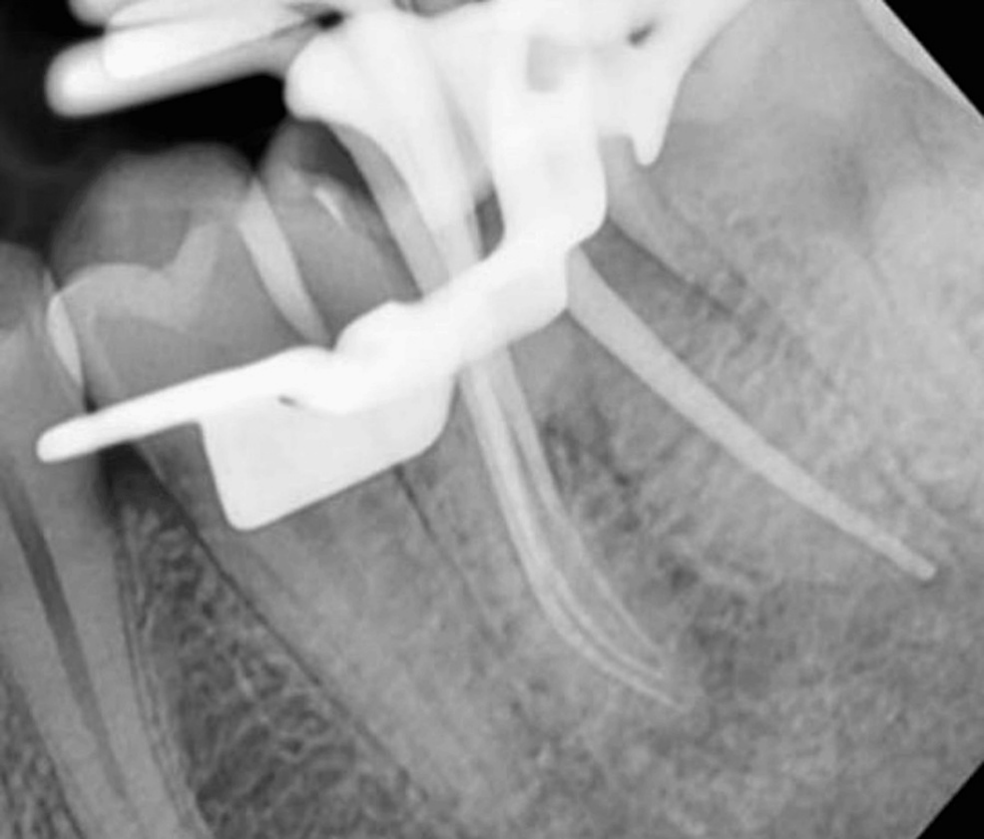
Mastercone fit assessment done using gutta-percha cones in the mandibular molar

The working length of the mesiobuccal and mesiolingual canals was both 21 mm, that of the mid-mesial canal was 21.5 mm, and that of the distal canal was 22 mm using a 10 Kerr file (Mani, Japan). Post-obturation radiography revealed that the mid-mesial canal was independent of the mesiolingual canal extending from the coronal to the apical area (Figure 9).

**Figure 10:**
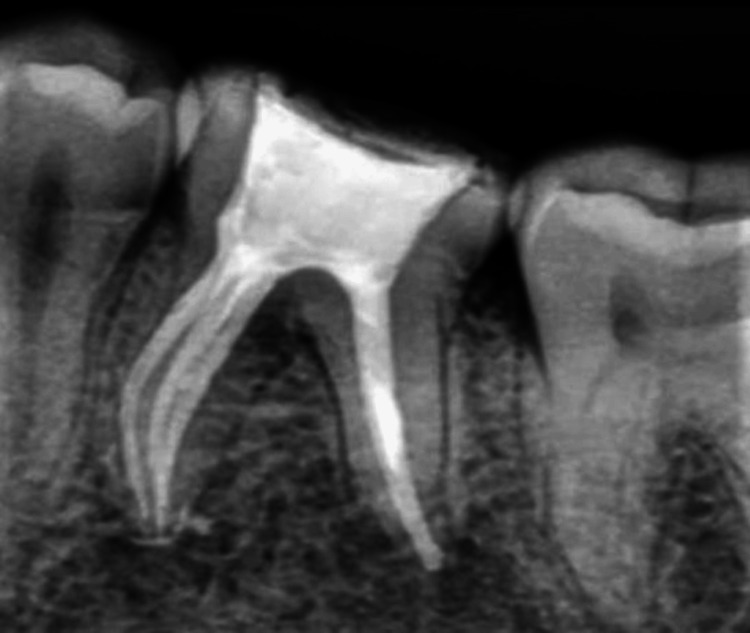
Obturation and post-endodontic restoration with resin composite

The access cavity was refurbished with composite restoration (3M ESPE, Maplewood, MN) (Figure 10).

## Discussion

This case highlights the need to consider and identify the mid-mesial canal system in lower molars using radio-visiographs, which were taken from the mesial as well. Fabra-Campos found that 2.6% of molars have three passages in their mesial root; merged canals are present in 1.7% of the population, with the mesiobuccal canal in the apical one-third region, and independent canal types are present in 1.6% of the population. The Indian population showed a prevalence rate of 29.7% for the mandibular first molar and 16% for the mandibular second molar [7]. Goel et al. documented the presence of mandibular first molars that showed three mesial canals in 13.3%, four mesial canals in 3.3%, and three distal canals in only 1.7% of the population [8]. Also, in terms of the number of apical foramina present, it was found that only a single apical foramina existed in 30%, two foramina in 60%, three in 6.7%, and four in 3.3% of the cases. Mesial roots of the first and second mandibular molar teeth tend to have a single large canal until the age of 11 and 15, respectively; secondary dentine deposits occur at the age of 30-40, highlighting the fact that the canal system in the root apex and the middle third region is completely established until that time period [9].

There is also a chance of large differences leading to reticular forms. Intercanal communication was low in the early and middle age group but was found to be high among the middle age group. Understanding these age-governed differences existing within the confines of the root canal system helps to locate and negotiate canals and then manage them [10]. Without a doubt, proper access cavities preparation is essential in locating the root canals. Several methods are employed in this respect and have been tried and routinely utilized in endodontic practice, such as the utilization of a sharp explorer examination of the pulp chamber floor, furrowing of grooves using an ultrasonic tip, staining of pulpal floor with 1% methylene blue dye indicator, and execution of a champagne bubble test using sodium hypochlorite, fiber-optic transillumination, and visualization of minute bleeding points on the pulpal floor [11].

From a clinical perspective, supplementary canal refers to continued bleeding in the tooth with signs of either the presence of pulpitis or bleeding from normal vital pulps, even after the completion of the entire biomechanical preparation of the tooth. Some of the important factors to look for in locating additional canals are apical rarefaction (nearly necrotic) in the root, tangential location of endodontic working length measurement, inconsistent readings shown on apex locators, torsion in the course of sinus tracts that bifurcate peripherally from the central canal, or a "catch" felt on the radicular canal wall during biomechanical preparation [12]. In a wide majority of cases, mid-mesial canals are concealed by dentinal projections along the mesial flange of pulpal chamber walls. Dentinal abundance is generally situated amid two foremost mesial canals. Ultrasonics offer a breakthrough in scouting and recognizing extra canals, eliminating the obstruction caused by conventional handpieces that obliterate vision.

The working tip of a particular ultrasonics is 10 times smaller than the size of the minutest round bur. The abrasive covering allows for the meticulous and gentle elimination of calcification and other obstructions over pulp chamber orifices. Dental loupes are among the recommended and popular magnification systems employed in endodontic routines. In this case series, dental loupes (Eighteeth, Brilliance, Changzhou Sifary Medical Technology Co., Ltd., China) were used to manage the first two cases endodontically. The dental operating microscope offers an added advantage in aiding for better illumination and superadded visibility. It detects delicate color changes, improves comprehension of dentinal floor mapping and passage of finer instrumentation, and provides coaxial illumination and excellent magnification. In this report, the middle mesial canals were located at equidistant positions flanked by two mesiobuccal and mesiolingual canals on either side. Inadequate diagnosis of radicular configurational anatomy and failure to recognize developmental abnormalities track into insufficient root canal debridement, resulting in poor endodontic results and subsequent need for retreatment or endodontic surgery [13]. This reinforces the prerequisite for thorough examination throughout treatment procedures to disinfect and reshape the system further effectively.

## Conclusions

Mid-mesial canals are complex to diagnose and treat, requiring a combination of diagnostic imaging, fine-tuned instruments, and careful obturation procedures. In this case report, we emphasize the significance of a multifaceted approach toward the treatment of middle mesial canals, which encompasses the measures of appropriate identification of pathological defects, negotiating anatomical curvatures and complexities in the root canal system, and ensuring the benefits of obtaining a three-dimensional seal. A confluence of these key features when kept in mind during the treatment of mid-mesial canals will help the clinician achieve comprehensive patient care and preserve the tooth for enhanced long-term prognosis.
